# Dr. James G. Van Marter

**Published:** 1901-12-15

**Authors:** 


### DR. JAMES G. VAN MARTER.

Dr. James G. Van Marter died at his home in Rochester, N. Y., October 21st, in his sixty-seventh year. He graduated from the Ohio Dental College and located for practice in Basle, Switzerland, where he practiced for six years, and then located in Florence, Italy, and afterwards in Rome. He was a successful practitioner and had high professional standing. For the last eight years he has resided in this country.

